# Health research systems in change: the case of ‘Push the Pace’ in the National Institute for Health Research

**DOI:** 10.1186/s12961-019-0433-2

**Published:** 2019-04-08

**Authors:** Rebecca Moran, Jennifer Butt, Simon Heller, Jeremy Hinks, Lynn Kerridge, Mark Samuels, Stephen Hanney

**Affiliations:** 1 NIHR Evaluation, Trials and Studies Coordinating Centre, Alpha House, Enterprise Road, Chilworth, Southampton, SO17 1BJ, United Kingdom; 2 Department of Oncology & Metabolism, University of Sheffield Medical School, Room EU38, E Floor, Beech Hill Road, Sheffield, S10 2RX, United Kingdom; 3 Medicines Discovery Catapult, Mereside, Alderley Park, Alderley Edge, Cheshire, SK10 4TG, United Kingdom; 4 Health Economics Research Group, Brunel University London, Uxbridge, United Kingdom

**Keywords:** Health research management, continuous improvement, productivity, National Institute for Health Research

## Abstract

**Background:**

Those running well-organised health research systems are likely to be alert for ways in which they might increase the quality of the services they provide and address any problems identified. This is important because the efficiency of the research system can have a major impact on how long it takes for new treatments to be developed and reach patients. This opinion piece reflects on the experience and learning of the United Kingdom-based National Institute for Health Research (NIHR) when it implemented continuous improvement activity to improve its processes.

**Discussion:**

This paper describes the structure and work of the NIHR and why, despite is successes as a health research system and ongoing local continuous improvement, it believed in the value of an organisation-wide continuous improvement activity. It did this by implementing an approach called ‘Push the Pace’. Initially, the organisation focused on reducing the amount of time it took for research to transition from an early concept to evidence that changes lives. This scrutiny enabled the NIHR to realise further areas of improvement it could make – additional goals were increased transparency, process simplification, and improved customer and stakeholder experience. We discuss our experience of Push the Pace with reference to literature on continuous improvement.

**Conclusion:**

Continuous improvement is a cycle, an activity that is done constantly and over time, rather than an act or linear activity (such as Push the Pace). We believe that the work of Push the Pace has initiated a strong commitment to a culture of continuous improvement in the NIHR. This is significant because culture change is widely recognised as immensely challenging, particularly in such a large and distributed organisation. However, our biggest challenge will be to enable all staff and stakeholders of the NIHR to participate in the continuous improvement cycle.

## Background

Those running well-organised health research systems are likely to be alert for ways in which they might increase the quality of the services they provide and address any problems identified. In the *Lancet* in 2009, the analysis of Chalmers and Glasziou presented a major challenge to all health research systems by claiming that 85% of all health research is avoidably ‘wasted’ because too much of the research asks the wrong questions, is badly designed, not published or poorly reported [1]. Further challenges appeared from the analysis showing how long it can take for new ideas to go through the various research stages and eventually lead to improved health policies, practices and health gain [2]. While some of the elapsed time is necessary to allow the research processes to take place and the safety of any new interventions to be thoroughly checked, there are also occasions when unnecessary delays occur [3]. It is desirable to reduce these delays, wherever it is practical and safe to do so, in order to bring improved treatments to patients more rapidly and increase the returns on public investment in research.

Subsequent analysis has considered how far a range of health research stakeholders had attempted to address the issues raised by Chalmers and Glasziou – it found progress was variable [4]. The National Institute for Health Research (NIHR) has developed an internationally recognised model to ensure that the research it funds answers the most important questions, is appropriately designed, efficiently delivered, unbiased, published in full, appropriately disseminated and useable. This model, which both pre-dates but also builds on the work of Chalmers and Glasziou [1], is kept constantly under review [5]. Some health research funders have looked to address the challenges they face by adopting a continuous improvement approach. For example, in the Irish Republic, the main health research funding body, the Health Research Board, explicitly stated in its new strategy in 2015 that it anticipated the collection of data would “*facilitate tracking of changing trends in the types of outcomes and impacts linked to the strategy and enable a process of continuous improvement in the services we provide*” ([6], p. 36). Continuous improvement initiatives put in place the necessary elements to enable an organisation to identify and implement improvements on an ongoing basis [7].

The term ‘continuous improvement’ refers to a number of methodologies that aim to improve efficiencies within production or service processes. The most well-known are Lean, Six Sigma and Total Quality Management. Continuous improvement, in the form of Lean, entered the management lexicon with the publication of The Machine that Changed the World [8]. This book outlined the principles behind Toyota’s successful manufacturing processes, which were concerned with reducing waste and enhancing value from the perspective of the customer [9].

Lean is sometimes associated with the elimination of waste [10] and can be described in five principles [8, 9], namely (1) identify value from the customer’s point of view, (2) identify the process that produces that value and eliminates wasted steps, (3) make service flow continuous, (4) introduce pull between all the steps where continuous flow is impossible, and (5) manage towards perfection so non-value adding activities will be removed such that the number of steps, time and information needed for service continually falls. The assumption behind these principles is that organisations are made up of processes and, through engaging with these principles in a sequential way, organisations can work to reduce waste, add value and continuously improve [11]. Continuous improvement describes an activity that must be done as a constant exercise and over time [12]. Although conceived within the manufacturing industry, continuous improvement methodologies have been used in public service organisations with varying outcomes (for example, in health providers [13, 14] and Her Majesty’s Revenue and Customs (HMRC) [15]).

In this Opinion piece, the authors draw on their respective experience of the leadership and involvement in the Push the Pace project to tell the story of a programme of improvement in a national health research system. Building on a series of iterations between team members, we describe and analyse what we did, why we did it, and the lessons we learnt. Our intention is to share our experience of change in order to promote the idea that those running health research systems can also scrutinise their own processes in an attempt to make continuous improvements. We begin by briefly outlining the NIHR in England, and explaining why we felt a programme of improvement was necessary. We then reflect on continuous improvement more generally and share our lessons learnt.

## Developing and applying improvements

### The National Institute for Health Research (NIHR)

The NIHR is funded through the Department of Health and Social Care to improve the health and wealth of the nation through research. Established in 2006, it aims “*to create a health research system in which the NHS* [National Health Service] *supports outstanding individuals, working in world-class facilities, conducting leading-edge research, focused on the needs of patients and the public*” ([16], p. 5). Today, the NIHR is the nation’s largest funder of health and care research [17]. Its inception marked a step change for health research in the United Kingdom, embedding an innovative national health research system in the NHS and focusing on patients’ needs [18]. Before this, there was an awareness that weaknesses in NHS research and development funding sometimes resulted in poor quality research, funding being diverted to support service delivery, and a decline in the number of clinical academics [19]. The Director of NHS Research and Development (and now Chief Medical Officer for England), Professor Dame Sally Davies, led the newly established NIHR to transform the research landscape for the benefit of patients and the public.

The NIHR also plays a key role in the Government’s strategy for economic growth, attracting investment by the life-sciences industry through its world-class infrastructure for health research. As well as patients and the public, the NIHR works in partnership with many sectors, including the NHS, public health, Government funders, the academic and third sectors, and industry. At the core of the aims of the NIHR is the commitment to improve lives (Box 1) [20].

**Fig. 1:**
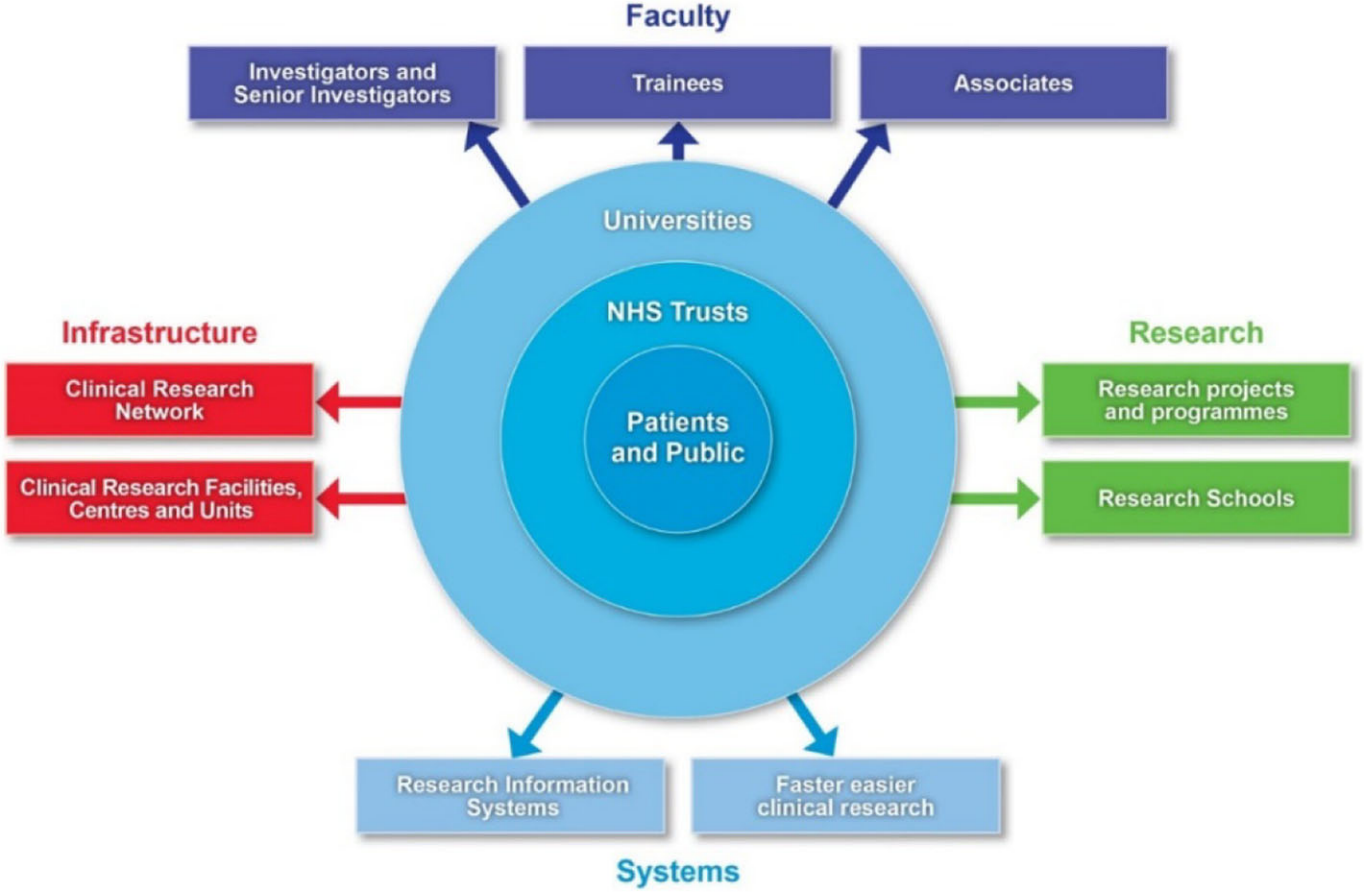
National Institute of Health Research (NIHR) health research system [20]. The NIHR manages its health research through four main work strands, namely infrastructure, faculty, research and systems; the interests of the patients and the public are at its core

The NIHR is a large multifaceted and virtual organisation whose components are spread over the country. It manages its health research through four main work strands (Fig. 1). The first is infrastructure, which provides the facilities and people for a research environment. The second is Academy (formerly known as faculty) to support individuals carrying out research. The third is the commissioning and funding of research itself. The fourth is systems, which describes unified and streamlined systems for managing research and its outputs.

This structure is federal in nature. As indicated in the diagram (Fig. 1), the NIHR is a mix of individuals, networks, teams, units, centres and systems. Right from the start there was interest in monitoring performance and a team of evaluation experts devised a framework that attempted to link “*early indicators of performance with longer-term research impacts*” [21]. Over the last 10 years, despite significant impact on the landscape of health research in the country [19], it became apparent that these multiple components were not easily able to transition a piece of research amongst themselves. That is to say, taking an idea from its earliest concept, such as a promising development in a laboratory, to being patient ready [22]. This is clearly important, because inefficiencies can ultimately delay new treatments reaching patients. A growing frustration, felt both by the NIHR and researchers, highlighted roadblocks within the NIHR in setting up, conducting research and disseminating findings. It was apparent to many involved with the NIHR that there was room to improve processes and speed up the pace of research that could bring benefits to patients and the public.

### Push the Pace

The story of Push the Pace begins with conversations. In 2014, Mark Samuels, a co-author of this paper, and then Managing Director of the NIHR Office for Clinical Research Infrastructure, attended a meeting with the United Kingdom government Cabinet Office at Cranfield School of Management. Kate Silver, then head of the Cabinet Office Continuous Improvement team, spoke broadly about Lean and the experience of HMRC in implementing it. At the Cranfield, Samuels explored with Silver whether such improvements could be made to a national organisation as large and distributed as the NIHR. He subsequently explored the idea with the CEO of the NIHR Evaluation, Trials and Studies Coordinating Centre (and also a co-author of this paper), Lynn Kerridge. The two of them went on to meet Silver and discuss how continuous improvement could benefit the parts of the NIHR that they led. Following up on the experience of HMRC, they also learnt from the Food and Environment Research Agency who had experience of continuous improvement activities. Particularly, Samuels and Kerridge reflected on the culture of the NIHR and the role a more collaborative leadership might play, both in drawing together the various components of the organisation and challenging the disaggregated culture of the organisation.

Kerridge and Samuels listened and learnt from the experiences of these other organisations and held a workshop exploring the progress of dementia research. Specifically, the journey a research idea takes to come to fruition as new evidence with the power to change patients’ lives. A similar conversation was then held at a meeting for diabetes research. This discussion highlighted ‘road blocks’ in the NIHR research funding and management process which prevented rapid establishment, conduct, dissemination and take up of research. Experts in diabetes and research management attended and the meeting identified areas where delays were present. David Sheldon, Continuous Improvement Manager at the Environment Agency, shared his experiences and the group mapped the research pathway and its stakeholders. They interrogated the journey a research topic takes from its earliest stage as an idea, to becoming new evidence with the potential to change practice. They identified handover points and pull through mechanisms to support the dissemination of research. This activity began the formalisation of Push the Pace. Its early aim was to identify areas for improvement across the whole of the health research pathway, to continually improve “*what we do and how we do it, and for this to make a real difference to patients’ lives*” [23]. This was made possible by high-level collaboration and the enthusiasm of other public sector organisations to share their experiences of continuous improvement.

When it was formalised as a programme of activity in 2014, the aim of Push the Pace was focused on time reduction (Box 2). It is highly desirable to reduce the time research takes wherever practical and safe to do so, in order to bring improved treatments to patients more rapidly and increase the returns on public investment on research [18, 24].

#### Push the Pace: the programme

The scope of Push the Pace was kept strictly to areas that the NIHR could influence. This was a key decision by Samuels and Kerridge. Throughout the project, there was often considerable pressure to increase the scope, which would have further complexity to an already complex programme.

**Fig. 2:**
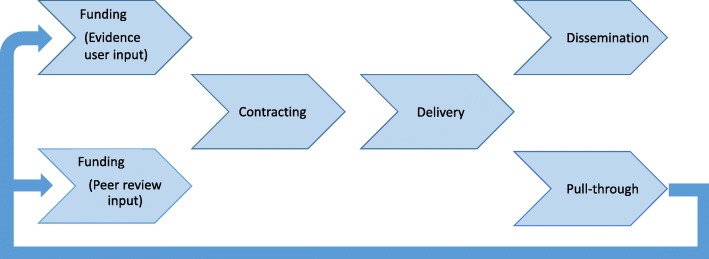
Work streams in Push the Pace [25]. The work streams corresponded to the journey a NIHR-funded research topic takes, from its identification as an important area of research through to its dissemination as evidence

Push the Pace identified six key areas and organised work streams to identify potential changes which could speed up health research [23]. Figure 2 shows these areas in their order within the research pathway [25]. This pathway refers to the management of research, wherein a research question is identified and a research application is approved and then a research team is awarded funding and a contract, managed by the NIHR, is agreed between the Department of Health and Social Care (the funder) and the research team. The NIHR monitors the progress of funded research, manages its delivery and helps facilitate the publication and dissemination of the research after it is completed.

Two work streams were concerned with the early stage of the research management process. First, the ‘evidence user input’ work stream looked at ways of increasing the involvement of the end user of research in making funding decisions; this was done in order to ensure that the research NIHR funds are of the highest value to evidence users. This work stream identified ways of making better use of existing expertise to fulfil this function. Secondly, the ‘peer review’ work stream explored how peer review of research funding applications could be proportionate such that both the quality of the review is high and the task itself is not overly onerous for reviewers. Ways to considerably improve consistency and better target peer review requests were identified, and as a result we expect to improve the rate of peer review response and the overall process [26]. The third work stream shown in Fig. 2 is ‘contracting’. After a project is approved for funding there can be delays in the arrangement of the contract and funds, leading to delays in the research project starting. The delays are in large part due to concerns about risk on both Department of Health and Social Care’s side (the risk of providing funds before a contract is signed) and the recipient’s side (the risk of proceeding before funds have been received). Push the Pace succeeded in implementing a ‘letter of intent’ and early indications are that this is speeding up the start of research studies before contracts are finalised.

After a research project starts there can be delays and challenges to its completion. In the ‘delivery’ work stream, we implemented changes to significantly improve study delivery to time and target and to reduce the number of study extensions. At the completion of a funded project it is essential that the findings are targeted to those for whom they are most relevant, since research results can only be taken up if the end user is aware of them. Through the ‘dissemination’ work stream we worked to improve the quality of dissemination materials and the targeting of dissemination activities. Finally, in the ‘pull-through’ work stream, we planned and piloted innovative mechanisms to speed up and improve the handover of research from the experimental parts of the organisation (such as NIHR Biomedical Research Centres [27]) into the different programmes on the NIHR research pathway.

All of these work streams had cross-NIHR leadership and involvement from across the organisation, and in some cases involvement and support from external stakeholders as well. In addition, the programme benefited greatly from an active and engaged advisory board of clinicians and academics, who were also supported by a lay member.

#### Push the Pace: what we learnt

We initially instigated Push the Pace with the single goal of reducing time but realised part way through that the programme could help us to achieve the additional goals of increased transparency, process simplification, and improved stakeholder experience. For example, it became clear to us that a lack of consistency across the different funding programmes was causing barriers for the research community. The different funding programmes used varying terminologies and management processes which the researchers applying for funding found confusing. The external perception was that we were ‘one’ organisation – the NIHR – but internally we operated more in ‘silos’, each centre or research programme with its own language and documentation and expert bureaucratic practices. Such silos are not unusual in large organisations. We realised the importance of simplification and coordination during Push the Pace and made significant changes towards realising a set of consistent processes across the organisation. We streamlined the application process for all NIHR research programmes. Furthermore, we realised the importance of furthering a sense of unity internally, so that our organisation continues to focus on and takes action to operate as ‘one NIHR’, shaping a culture of consistency and unity amongst our staff and procedures. This is significant, because it showed that fostering a sense of unity and support for major change are possible in a complex, national organisation. Important success factors were leadership shouldering the burden of risk, minimising hierarchy and empowering people at all levels within the organisation to act.

A second realisation as a result of Push the Pace was that the NIHR places a high value on process (by this we mean our practices, our ‘business as usual’ way of managing research). Unintentionally, this emphasis increased the burden for both the research community and the NIHR staff. As a custodian of public money, our emphasis on process was intended to help reduce risk. However, we recognised a better balance was needed between risk and process in our practices in order to realise our goal of improved stakeholder experience. Two examples of this are the improvements made to the contracting and the peer review processes. This is significant, because it demonstrated that a government organisation – traditionally thought to be risk averse – can strike the right balance between risk and process.

In total, the Push the Pace programme lasted almost 4 years, from spring 2014 until the end of 2017. In reflecting on Push the Pace we found our thinking was mirrored in the literature about continuous improvement. The following section contextualises our learning within a broader discussion on continuous improvement.

### Continuous improvement

There is no theoretical basis for continuous improvement [28]. It is a general term that has acquired many of its attributes from quality initiatives such as Total Quality Management and Lean manufacturing. For successful continuous improvement cycles, Sanchez and Blanco [12] argue that there need to be three factors in play. Firstly, the recognition that continuous improvement is a cycle, not only an ‘act’; it is a constant activity that must be done over time. Our challenge is to take our learning from Push the Pace, which was a programme of work designed to find areas in which we could improve our practices, and transition to a culture of continuous improvement within the organisation, a culture that is not dependent on a codified improvement ‘programme’ or ‘project’.

Secondly, Sanchez and Blanco [12] state that all people in an organisation should participate. This is a challenge for two reasons. The first is due to the size and structure of the NIHR, spread as it is across England. We needed to work out how to integrate continuous improvement strategies across the whole organisation. We did this by networking with a wide range of stakeholders, inside and outside of our organisation for Push the Pace. This included many parts of the NIHR, a number of NHS Trusts, higher education institutions and charities, and our Advisory Board to name but a few. We received senior endorsement for what we were trying to achieve from both our sponsoring stakeholders, the NIHR Strategy Board and the Department of Health and Social Care. The second challenge was how we could shorten and improve the processes we use to manage research. This required the involvement of our research management staff whose day-to-day work is busy and time sensitive. The nature of their roles meant they had restricted capacity to make the time for work like Push the Pace that is important, but does not directly relate to immediate goals. During Push the Pace only two of the 14 work stream co-leads were given protected time to work on the programme. We are still considering the challenge – how can we continuously improve and manage our business as usual? The NIHR needs to engage its whole workforce with a sense of efficacy and commitment to continuous improvement in their day-to-day activities.

Thirdly, and obviously, the aim of continuous improvement is to improve [12]. Therefore, the organisation should focus on reducing unnecessary practices and identifying new areas for improvement. We achieved the former in that, for example, we have reduced the time of our contracting processes and we have better targeted dissemination activities. We also achieved the aim of identifying new areas for improvement in that, although our initial focus was to save time, we identified that we could also enhance the experience of our stakeholders and simplify our processes.

Radnor and Osborne [29] discuss the relationship between customer focus and process focus. They argue that, if the focus is only on process (which they describe as efficiency) then service (effectiveness) may be compromised. Similarly, by focussing only on the customer, inefficiencies or waste may be built into the process. Radnor and Osborne argue that only by focusing on both efficiency and effectiveness will sustained improvements be achieved. This is confirmed by a key learning from Push the Pace, namely that we had previously prioritised our processes over the experience of our applicants. Radnor and Johnston’s work in the context of other public sector organisations identify a challenge in “*the ability to create a link between internal operations, service delivery and customer satisfaction and value*” ([13], p. 912). We feel it is vital that adherence to our core aim – to change the lives of patients and the public for the better through research – must be understood throughout the NIHR, including its whole workforce and stakeholders, in order for a culture of continuous improvement to take shape and benefit our practices and business as usual. Radnor and Johnston continue: “*Indeed, if the public sector does not or is not able to develop understanding of the value for the citizen/market as a driver any further development of Lean or process improvement may not be sustainable. This may mean that the agenda may always be on efficiency rather than effectiveness and indeed take a goods/production rather than service dominant logic*” ([13], p. 912). Normann [30] refers to a ‘virtuous circle’ of service improvement. This circle explicitly links improved performance within an organisation to improved performance with service users. Radnor and Osborne [29] propose that the focus of Lean (or continuous improvement as we define it more broadly) must be driven by addressing the issue of how to add value to the lives of the end-users of public services. Push the Pace taught us that there are further ways for the NIHR to achieve this, in addition to aiming to increase the speed of the research process.

While we are not aware of any exactly parallel initiatives from other funders, we are aware that, in the context of concern about the waste in research noted above [1, 4], there is growing interest from health research funders in how best to ensure value in health research [31]. In particular, the research funders who came together as the Ensuring Value in Research Funders’ Collaboration and Development Forum agreed a consensus statement at their meeting in June 2017 that recognised their responsibility “*to advance the practices of health-related research and research funding*” [31]. Convened by the NIHR, the Netherlands Organisation for Health Research and Development, and the Patient-Centered Outcomes Research Institute in the United States of America, the Forum has a growing international membership [32]. Its meetings might present opportunities to discuss the findings from the Push the Pace project in relation to developments in other systems, such as the reference to continuous improvement in the current strategy from the Irish Health Research Board [6], a member of the Forum. The scale of funding for research means that improvements in efficiency matter. The NIHR invests £1 billion per year in research; therefore, even a small percentage reduction in waste makes a considerable difference.

## Conclusion

Ultimately, the end-users of the NIHR are patients and the public. Therefore, every agent in the production of research is part of a process that should enable lives to change for the better. Continuous improvement is a cycle, an activity that is done constantly and over time, rather than an act or linear activity. We believe that the work of Push the Pace has initiated a strong commitment to a culture of continuous improvement in the NIHR. However, our biggest challenge will be to enable all staff and stakeholders of the NIHR to participate in the continuous improvement cycle. If the end goal of health research is to improve the lives of patients and the public, there is a sense that every agent involved in the production of research, from its concept to its implementation in practice, has a responsibility in their day-to-day work to identify new areas of improvement and remove obstructions, ultimately to ensure lives are changed for the better. We hope that a formal evaluation of the Push the Pace approach will be possible in the future, but for now, this Opinion piece provides initial lessons for those running health research systems about how they can scrutinise their own processes in an attempt to make continuous improvements. We hope this encourages them!

Box 1 Aims of the NIHR [20]

**Table:** 

• Establish the NHS as an internationally recognised centre of research excellence	
• Attract, develop and retain the best research professionals to conduct people-based research	
• Commission research focused on improving health and social care	
• Strengthen and streamline systems for research management and governance	
• Increase the opportunities for patients and the public to participate in, and benefit from, research	
• Promote and protect the interests of patients and the public in health research	
• Drive faster translation of scientific discoveries into tangible benefits for patients	
• Maximise the research potential of the NHS to contribute to the economic growth of the country through the life sciences industry	
• Act as a sound custodian of public money for the public good

Box 2 Push the Pace: summary of key lessons

**Table:** 

• The NIHR began an activity of continuous improvement; its initial goal was to reduce the amount of time it took for research to transition from an early concept to evidence that changes lives	
• As the activity of Push the Pace was underway, we realised further improvements were possible:	
○ Increased transparency and simplification of our research management processes	
○ Improved customer and stakeholder experience	
○ Commitment to a culture of consistency and unity amongst NIHR staff and procedures

## Abbreviations

HMRC: Her Majesty’s Revenue and Customs
NHS: National Health Service
NIHR: National Institute for Health Research
